# New records of native and introduced fish species in a river basin of Western Ecuador, the Chocó-Darien Ecoregion, using DNA barcoding

**DOI:** 10.1371/journal.pone.0298970

**Published:** 2024-03-08

**Authors:** Daniel Escobar Camacho, Karla S. Barragán, Juan M. Guayasamin, Gabriela Gavilanes, Andrea C. Encalada

**Affiliations:** 1 Laboratorio de Ecología Acuática, Instituto BIOSFERA, Universidad San Francisco de Quito, Quito, Ecuador; 2 Laboratorio de Biología Evolutiva, Instituto BIOSFERA, Universidad San Francisco de Quito, Quito, Ecuador; DePaul University, UNITED STATES

## Abstract

DNA barcoding, based on mitochondrial markers, is widely applied in species identification and biodiversity studies. The aim of this study was to establish a barcoding reference database of fishes inhabiting the Cube River from Western Ecuador in the Chocó-Darien Global Ecoregion (CGE), a threatened ecoregion with high diversity and endemism, and evaluate the applicability of using barcoding for the identification of fish species. Barcode sequences were obtained from seven orders, 17 families, 23 genera and 26 species, which were validated through phylogenetic analysis, morphological measurements, and literature review. Our results showed that 43% of fish species in this region are endemic, confirmed the presence of known species in the area, and included the addition of three new records of native (*Hoplias microlepis*, *Rhamdia guatemalensis* and *Sicydium salvini*) and an introduced species (*Xiphophorus maculatus*) to Ecuador. In addition, eight species were barcoded for the first time. Species identification based on barcoding and morphology showed discrepancy with species lists from previous studies in the CGE, suggesting that the current baseline of western fishes of Ecuador is still incomplete. Because this study analyzed fishes from a relatively small basin (165 km^2^), more molecular-based studies focusing on fish are needed to achieve a robust sequence reference library of species inhabiting Western Ecuador. The new sequences of this study will be useful for future comparisons and biodiversity monitoring, supporting the application of barcoding tools for studying fish diversity in genetically unexplored regions and to develop well-informed conservation programs.

## Introduction

The Chocó-Darien Global Ecoregion (CGE) is a mountainous lowland area in the Pacific coast expanding from Eastern Panama and Western Colombia to Northwestern Ecuador [1]. The uplift of the northern Andes, peaking around 23 mya [2], isolated the CGE from the Amazon system. Groups of endemic species emerged producing a significant impulse of diversification, thus, this region is classified as one of the 25 biodiversity hotspots in the world due to its unique and threatened biodiversity [3]. The CGE is formed by four terrestrial ecoregions: Choco-Darien Moist Forests, Magdalena Urabá Moist Forests, Western Ecuador Moist Forests, and smaller sections of Eastern Panamanian Montane Forests. The CGE is one of the rainiest regions on Earth, with annual precipitation up to 13 000 mm in some areas [4]; however, several river networks can be intermittent or ephemeral in this region, driven primarily by the seasonal stark rainfall variation [5–8].

Despite of being an area with high priority for conservation due to its high endemism and ecosystem services, the Western Ecuador Moist Forests have suffered extensive and rapid land transformation in the last decades [9, 10]. The most important drivers of deforestation and degradation are agriculture and cattle grazing with higher rates of deforestation concentrated around buffer zones of private and public reserved areas, including the ones of the Ecuadorian CGE [11]. As land-use transformation and climate change threaten diversity and the provision of ecosystem services from aquatic ecosystems in the Ecuadorian CGE, improving species knowledge is necessary to develop recommendations for their management, conservation, and restoration efforts.

The CGE harbors 23 major watersheds, which altogether harbor 264 freshwater fish species [12]. These assemblages include estuarine amphidromous species like gobies and mullets that migrate upstream to several Andean rivers [8]. The ichthyofauna of Western Ecuador is characterized by its isolation, high levels of endemism and relatively low richness when compared to the Ecuadorian Amazon (113 spp Western Slope *vs* 744 spp Napo basin alone) [13, 14]. Approximately 38% of freshwater fishes of the Ecuadorian CGE are endemic [15]. Fish studies in the CGE have characterized its diversity patterns, richness, adaptations and ecology [8, 16–19]. However, few studies have used molecular techniques focused on species-level identifications, systematics, and taxonomic diversity of CGE basins. Molecular research in CGE fish is imperative due to the challenging nature of species identification. This difficulty arises from the presence of species groups distributed in both trans- and cis-Andean basins (disjunct distributions) [20], the presence of cryptic diversity [21, 22] and because CGE’ fish have been less studied when compared to species from more diverse regions such as the Amazon.

Fish DNA barcoding is a useful primary tool for identifying fish species in studies that aim at characterizing fish diversity in specific areas [23–25]. However, it’s appropriate application can be precluded by the poor knowledge of the studied ichthyofauna and by incomplete sequences libraries and specimens’ inventories. This is because results from molecular findings always need to be validated by morphological analyses and species records throughout current and localized literature. Propper application of DNA barcoding leads to the development of comprehensive reference sequence libraries of taxonomic-curated species [26]. The present study tested the utility of DNA barcoding approach as a molecular technique for the identification of fishes within a region of the Ecuadorian CGE that has not been characterized at the molecular level before. Previous studies using molecular tools have proven to be highly informative for the Ecuadorian ichthyofauna. Studies have uncovered the presence of different lineages of wide-distributed species [27], the presence cryptic diversity [28], the rapid divergence of species inhabiting in different habitats [29] and even it has led to the discovery of new species [30]. By performing species identification through integrative analyses that included DNA barcoding, morphological measurements, and literature review from the Ecuadorian CGE, we evaluated the species identification success of this framework in a specific region of the Ecuadorian CGE. We predicted that 1) barcoding analyses would reveal previously undetected species in this region and that 2) this analysis would complement previous studies of the western fishes of Ecuador. Results of this research could contribute with useful information that will aid in conservation planning and aquatic habitat restoration in the CGE. Here we make a regional contribution to the study of CGE ichthyology, by characterizing the molecular barcodes of 26 fish species from the Cube River basin, an intermittent river from the Ecuadorian Chocó ecoregion.

## 2. Materials and methods

### 2.1 Study site

**Fig 1:**
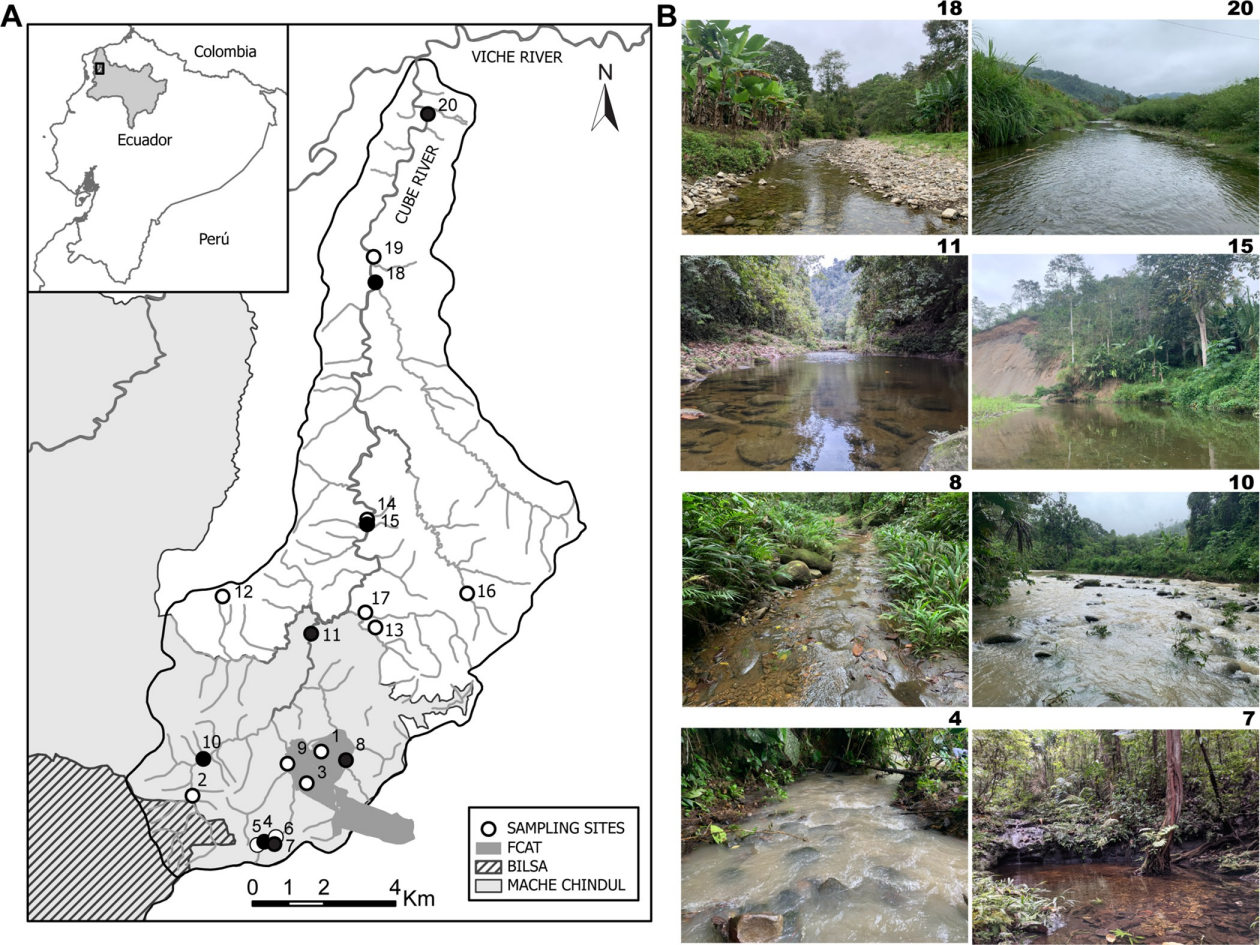
The Cube River basin (A) Map of the Cube River basin, within the Esmeraldas River basin (shown in the inset) showing 20 sampling sites across an altitudinal gradient (higher-south to lower-north). (B) Pictures depicting the aquatic ecosystems of several sampling sites. The numbers of black circles correspond to numbering of sampling sites on the right. Reprinted with permission from Jose R Daza, 2023.

The Cube River basin (169 km^2^ drainage area) is a seasonally intermittent river system that flows from South to North and is a tributary of the Esmeraldas River basin. The Cube River is in the northern Ecuadorian CGE where it flows into the Viche River at the northwestern side, and limits with the Mache-Chindul Reserve ridge at the east, and with the Bilsa Biological Reserve at the south (Fig 1). Approximately half of the headwaters of the Cube River basin overlap with the Mache-Chindul Ecological Reserve (REMACH), Fundación para la Conservación de los Andes Tropicales Reserve (FCAT), and Bilsa Biological Reserve. Despite of the evidently land-cover under management (Fig 1A), conservation entities in the Cube River share territories with farmers under varying types of agriculture and cattle grazing activities. The headwaters of the Cube River basin are within the private protected reserves (i.e., FCAT and Bilsa) that maintain the last remnants forests of CGE in Ecuador. At the Cube basin, 21 species of freshwater fish have been reported to occur, of which 43% are endemic. Recent investigations have shed light on the impact of a fragmented landscape on both, species richness and abundance, as well as the seasonal dynamics shaping communities in Laguna del Cube—a RAMSAR lake from the region [16, 31].

### 2.2 Sampling and ethics statement

Fish were sampled in 20 sampling sites along an altitudinal gradient (S1 Table in S1 File) in 2021. Fishing was conducted across rivers of varying widths, ranging from 1 m to 10 m. The methods employed included the use of hand, stop and seine nets (5 mm mesh size, 6 m or 10 m long, and 2 m high). Sampling was performed along zig-zag transects across the streams, while walking at a constant speed. Both ends of the transect were blocked with seine nets. All caught fish were maintained in a bucket until a few ones were selected for collection and tissue extraction, the remaining fish were freed in the same location. Criteria for selecting fish were adult size and if the species was rare. Common species were those known to inhabit the area, consistently found across multiple sites and in abundance during sampling. In contrast, rare species typically consisted of a single individual, and their identification was uncertain at the field site. Selected fish were transported to FCAT station where they were photographed and subsequently anesthetized and euthanized with eugenol oil. For some species, several individuals were abundant; however, other species were rare and only a few individuals were collected. Muscle and fin clip tissue samples were obtained and immediately preserved in 96% ethanol for DNA extraction. All whole fish samples were stored as voucher specimens in 70% EtOH after fixation in a 10% formaldehyde solution and deposited in the Ichthyological Collection of the Natural History Museum Gustavo Orcés V. at Escuela Politécnica Nacional (Ecuador), vouchers MEPN-I 19713–19761. This research complied with the requirements established in the Environmental Unified-Text-of-Secondary-Legislation regarding animal welfare laws, guidelines and policies approved by the Ministry of Environment, Water and Ecological Transition (Ministerio de Ambiente, Agua y Transición Ecológica del Ecuador—MAATE), which authorized scientific research and access to sampling sites through the permit (MAAE-ARSFC-2020-1057) and access to genetic resources through the permit (MAATE-DBI-CM-2023-0289).

### 2.3 DNA extraction, PCR amplification and sequencing

Two molecular markers corresponding to two partial genes from mitochondrial DNA were used to identify species: cytochrome c oxidase I (COI) and ribosomal 16S rRNA (16S). We used COI because, since its proposal [32], it has become the standard marker for animal identifications [33] and because fish from the CGE have been previously analyzed with this marker [27, 34–36] as well as with 16S [21, 22, 37]. For both markers, data from previous studies were readily available for comparisons (Genbank: https://www.ncbi.nlm.nih.gov/genbank/).

Genomic DNA was extracted from tissue using a cost-effective protocol for DNA isolation based on Guanidine Isothiocyanate [38]. Total DNA was extracted from a fin clip or muscle tissue using DNeasy Blood & Tissue Kit–Qiagen.

Polymerase Chain Reaction (PCR) amplification of 16S and COI genes fragment were performed in 25 μl reactions using 0.5 μl deoxynucleotide triphosphate (dNTP) (10mM), 2.5 μl PCR buffer 5X (200 mM Tris-HCl (pH 8.4), 1.5 μl MgCl_2_ (50 mM), 0.5μl of each primer (10 μM), 0.25 μl of Taq DNA polymerase, 1 μl template DNA [100 ng/ul] and 18.25 μl of H2O. Primers and PCR amplification protocols are presented in S2 and S3 Tables in S1 File, respectively. PCR products were visualized in agarose gels. Primers and dNTPs were then removed using ExoSap purification (ExoSap-it, GE Healthcare) for subsequent Sanger sequencing at Macrogen Inc, Seoul, Korea.

### 2.4 Genetic analysis and species identification

Once samples were sequenced, we compared them with available sequences of species of interest to analyze whether there were unrecorded species in the northern Ecuadorian CGE. Morphological measurements, predominantly focusing on body length and body depth, were conducted alongside an examination of their relationships with head length, fin placement, eye and snout position, and peduncle. Additional observations encompassed various meristic characters, including the number of teeth and scales, reinforced by taxonomic identification for all species. Further, to complement knowledge gaps in fish diversity, we compared our identified species with lists from the region by Navarrete-Amaya et al 2021 and Jiménez-Prado 2015 [13, 17].

We used MAFFT [39] to align nucleotide sequences and visual inspections of alignments, sequence editing and sequence assembly were done with Geneious Prime (Version 2021.1.1). For both genes, insertions and deletions were examined through visual inspection. Additionally, for COI, the presence of stop codons was checked using the “Translation” tool in Geneious. We generated two alignments for each gene, one that contained only samples from the Cube River basin and a second that contained external gene sequences from related species available in GenBank (S4 Table in S1 File). For the Cube River species alignment, we used MEGA [40] to analyze statistics such as pairwise identity (percentage of identical position between pairs of sequences) and proportion of identical sites (percentage of identical nucleotides across all sequences), and to obtain genetic distance with Kimura 2-parameter and p-distance between samples. Later, we trimmed both ends of the Cube River species alignments and both genes were concatenated into a single alignment. For the concatenated alignment some individuals were excluded because one of either 16S or COI were not amplified. For the second alignment, comparative species were chosen based on availability of sequences, species distribution and evolutionary relationships (same genus or species). Also, we sequenced additional samples of COI of *Pseudochalceus lineatus*, *Trichomycterus* spp and *Astroblepus* spp, because we noticed that these species exhibited exclusive distribution in isolated headwaters (S1 Fig in S1 File) and could be prone to isolation of metapopulations due to intermittency.

We built three phylogenetic trees: one for each gene (16S and COI) and one from the concatenated alignment. We used W-IQ-TREE [41] web interface for phylogenetic analysis where the tool searched for model selection and best tree search and support computation (1000 ultrafast bootstrap replicates [42] and SH-aLRT [43]) (S5 Table in S1 File). For the concatenated alignment, a partition file was created to specify the position of each gene and subsequently used in IQ-TREE for model selection for each gene. To classify species we analyzed where our samples would cluster and their position in reference to the sequences in GenBank [44].

For morphological classification, fish were identified to species level or the lowest possible taxonomic unit, using available literature from the Western fishes of Ecuador [13, 17] and updated revisions of representative groups in this region [29, 30, 45–47]. Because diagnostic morphological characters vary greatly with taxa, we evaluated different morphological characters for each specific group (S6 Table in S1 File) and we took measurements with a digital caliper (to the nearest 0.1 mm), following the methodology described by Weitzman and Fink [48], noting different aspects of each species. Fish species were identified through an integrative approach by following morphological taxonomic keys, analyzing records in the literature, and then incorporating the suggested IDs based on GenBank and phylogenetic relationships.

## 3. Results

**Fig 2:**
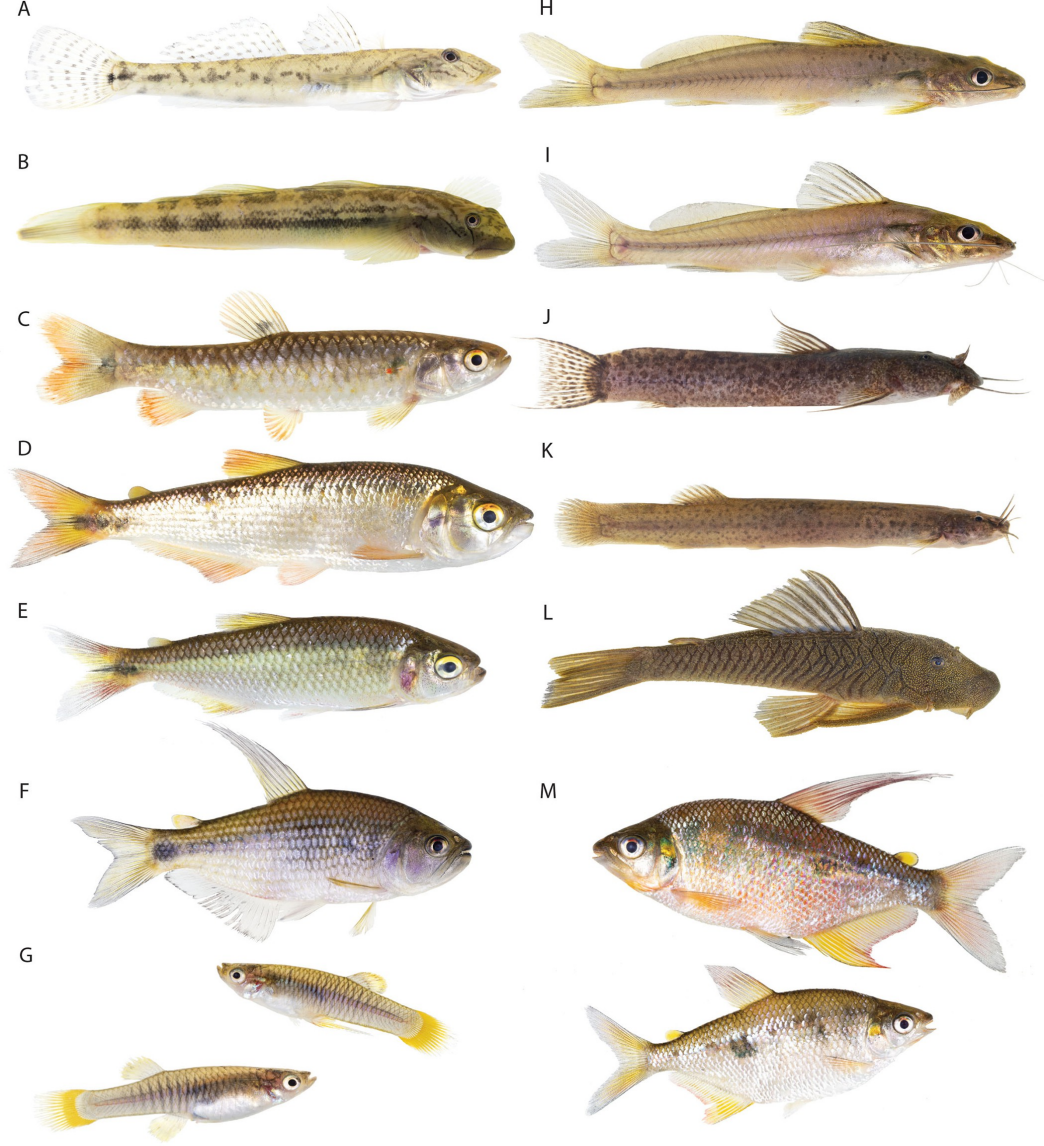
Freshwater fish of the Cube River basin. (A) *Awaous transandeanus*, (B) *Sicydium salvini*, (C) *Lebiasina bimaculata*, (D) *Brycon atrocaudatus*, (E) *Eretmobrycon* cf. *ecuadoriensis*, (F) *Pseudochalceus lineatus*, (G) *Peseudopoecilia fria* (male top, female below), (H) *Rhamdia guatemalensiss*, (I) *Pimelodella modestus*, (J) *Astroblepus* aff. *mindoensis*, (K) *Trichomycterus* aff. *banneaui*, (L) *Chaethosstoma bifurcum*, (M) *Rhoadsia minor* (male top, female below). Photos: courtesy of Karla Barragán.

A total of 26 freshwater fish species, 23 genera, 17 families from 7 orders were collected and analyzed in this study from 20 sampling sites along the Cube basin (Fig 2 and S2 and S7 Tables in S1 File). Three species are introduced while the remaining 23 constitute 20% of the fish reported for Western Ecuador [15] and 42.8% are endemic. Four species are amphidromous (*Awaous transandeanus*, *Sicydium salvini*, *Gobiomorus maculatus* and *Agonostomus monticola*) and records of three native species were unique to one sampling site (*Synbranchus marmoratus* and *Pseudocurimata boehlkei*, *Astroblepus cyclopus;* sites 10, 13 and 10 respectively (S1 Table in S1 File)).

### 3.1 Amplification and sequencing

A total of 111 mitochondrial barcode sequences of COI (68) and 16S (43) were obtained from 23 genera, 17 families, 7 orders of fishes (Table 1, S4 Table in S1 File). Although we performed several attempts for PCR amplification, no COI amplicons were obtained from *G*. *maculatus*.

**Table 1:** Species identity of specimens collected in this study. Identifications are based on molecular and morphological characters and relevant literature of species occurrence in Western Ecuador. Numbers in parenthesis indicate the number of analyzed specimens using morphology.

This study	Cube River basin species^†^	Esmeraldas River basin species^‡^
*Agonostomus monticola* (4)	Not reported	*Agonostomus monticola*
*Andinoacara rivulatus* (7)	*Andinoacara blombergi*	*Andinoacara blombergi* *A*. *rivulatus* *A*. *sapayensis*
*Astroblepus aff*. *mindoensis* (9) *Astroblepus cyclopus* (1)	*Astroblepus* cf. *fissidens*	*Astroblepus cyclopus* *A*. *eigenmanni* *A*. *fissidens* *A*. *grixalvii* *A*. *longifilis* *A*. *mindoensis* *A*. *whymperi*
*Awaous transandeanus* (6)	*Awaous transandeanus*	*Awaous transandeanus*
Brycon atrocaudatus* (11)	*Brycon dentex*	*Brycon alburnus* *B*. *dentex*
*Astyanax festae* (11)	Not reported	*Astyanax festae* *A*. *ruberrimus*
*Eretmobrycon cf*. *ecuadoriensis* (11)	*E*. *ecuadoriensis* *E*. *dahli*	*Eretmobrycon brevirostris* *E*. *dahli* *E*. *ecuadoriensis*
*Chaetostoma bifurcum* (8)	*Chaetostoma aequinoctiale*	*Chaetostoma bifurcum* *C*. *fischeri* *C*. *marginatum*
*Gobiomurus maculatus* (5)	Not reported	*Gobiomurus maculatus*
*Hoplias microlepis* (3)	*Hoplias malabaricus*	*Hoplias malabaricus* *H*. *microlepis*
*Lebiasina bimaculata* (8)	*Lebiasina bimaculata*	*Lebiasina bimaculata* *L*. *astrigata*
*Mesoheros festae* (3)	*Mesoheros festae*	*Mesoheros festae*
*Oreochormis niloticus* (1)	Not reported	*Oreochromis niloticus* *O*. *mossambicus*
*Pimelodella modestus* (5)	*P*. *grisea*	*Pimelodella modestus* *P*. *elongata* *P*. *grisea*
*Poecilia reticulata* (4)	Not reported	*Poecilia reticulata*
*Pseudochalceus lineatus* (10)	*Pseudochalceus boehlkei*	*Pseudochalceus lineatus*
*Pseudocurimata boehlkei* (2)	Not reported	*Pseudocurimata boehlkei*
*Pseudopoecilia fria* (11)	*P*. *fria*	*Pseudopoecilia fria*
Rhamdia guatemalensis* (7)	*R*. *quelen*	*Rhamdia cinerascens*
*Rhoadsia minor* (13)	*Rhoadsia altipinna*	*Rhoadsia altipinna* *R*. *minor*
Sicydium salvini* (2)	Not reported	*Sicydium hildebrandii* *S*. *rosenbergii*
*Synbranchus marmoratus* (1)	Not reported	*Synbranchus marmoratus*
*Trichomycterus* aff. banneaui (8)	*Trichomycterus spilossoma*	*Trichomycterus taenia*
*Trichomycterus* sp (3)		
*Xiphophorus maculatus* (5)	Not reported	Not reported
Not reported	*Batrochoglanis transmontanus*	*Batrochoglanis transmontanus*
Not reported	*Rineloricaria jubata*	*Rineloricaria jubata*

^†^Based on literature of the Cube River basin [16, 31].

^‡^Based on the fish species distribution of the Western Fish from Ecuador [13, 17].

*Species that match morphologically a species that has been reported to not inhabit in the area.

### 3.2 Alignments and genetic distances

The COI and 16S alignments had a mean length of 667 and 628 bp, respectively. For both genes, the aligned sequences contained no insertions or deletions, and COI sequence did not exhibit stop codons, indicating that all amplified sequences were functional. The average number of identical sites was higher in COI dataset whereas pairwise identity was higher in 16S (S8 Table in S1 File). The base composition showed that the average A content was the highest for both genes whereas average C and G content was the lowest in COI and 16S, respectively. Subsequently, alignments were concatenated in a single alignment of 1309 bp length containing 37 sequences with 45.5% of identical sites and a pairwise identity of 77.9%.

The K2P distance for COI sequences ranged from 0 to 32% with an average of 24%, whereas for the 16S dataset distances ranged from 0 to 30% with an average of 19%. More conserved values were obtained by p-distance method with a mean distance of 20% and 16% for COI and 16S, respectively (S9 Table in S1 File).

### 3.3 Genetic analyses and species identity

**Fig 3:**
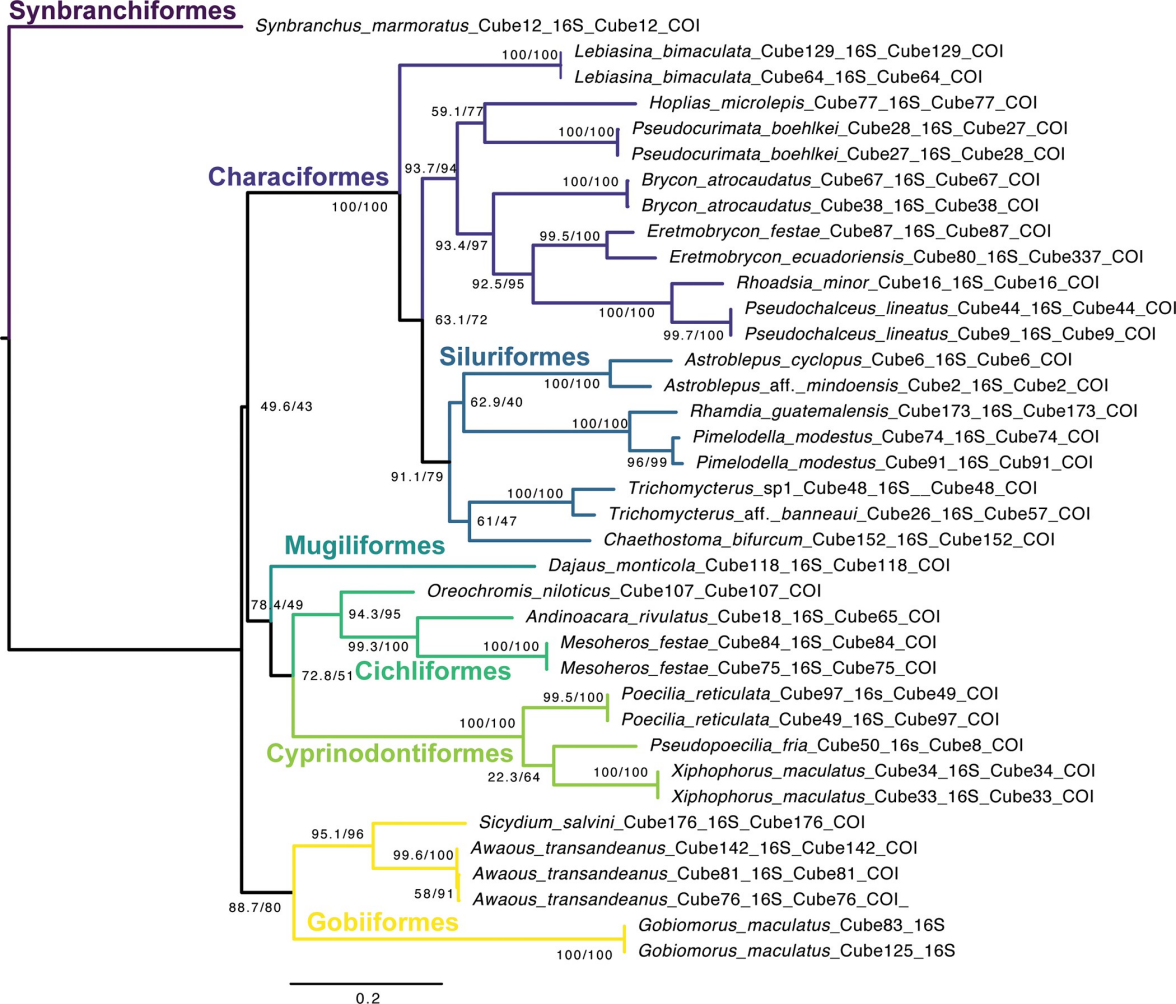
Phylogenetic relationships of fishes from the Cube River basin. Maximum likelihood phylogenetic tree based on partial 16S rRNA (16S) and c oxidase subunit I (COI) sequences (1309 bp), representing 26 fish species from the Cube River basin. Node statistical support is shown as: SH-aLRT support (%) / ultrafast bootstrap support (%).

The maximum likelihood tree of the Cube River species, including all 26 species is provided in Fig 3. All species formed distinct clusters in the tree and whenever there were duplicates of the same or congeneric species they clustered together within the same orders. Only *Lebiasina bimaculata* (Lebiasinidae: Characiformes) diverged earlier of the clade composed of Characiformes and Siluriformes (Fig 3). From 12 species we did not obtain any close match for both genes from molecular databases, whereas for 12 and 13 species we did not obtain a match for COI and 16S records separately, respectively (Table 2). Nine species (*Brycon atrocaudatus*, *Eretmobrycon cf*. *ecuadoriensis*, *Trichomycterus* sp1, *Trichomycterus* aff. *banneaui*, *Astroblepus* aff. *mindoensi*s, *Astroblepus cyclopus*, *Lebiasina bimaculata*, *Pseudopoecilia fria*, and *Pseudochalceus lineatus*) were barcoded for the first time.

**Table 2:** Blast match identity between 99–100% of sequenced species from the Cube River basin with available data from GenBank.

Species	COI	Close match	16S	Close match
*Agonostomus monticola*	X	MG496130.1	X	JQ060648.1
*Andinoacara rivulatus*	X	KU692246.1	X	LC009435.1
*Astroblepus aff*. *mindoensis*	-	NA	-	NA
*Astroblepus cyclopus*	-	NA	-	NA
*Awaous transandeanus*	X	MZ130161.1	X	MF927489.1
*Brycon atrocaudatus*	-	NA	-	NA
*Eretmobrycon brevirostris*	X	KF210042.1	X	KF209716.1
*Eretmobrycon* cf. *ecuadoriensis*	-	NA	-	NA
*Chaethostoma bifurcum*	-	NA	X	KP959851.1
*Gobiomurus maculatus*	NA	NA	X	KF415378.1
*Hoplias microlepis*	-	NA	-	NA
*Lebiasina bimaculata*	-	NA	-	NA
*Mesoheros festae*	X	DQ119216.1	X	DQ119187.1
*Oreochormis niloticus*	X	MK955804.1	X	MH567058.1
*Pimelodella modestus*	-	NA	-	NA
*Poecilia reticulata*	X	JX968695.1	X	KX816038.1
*Pseudopoecilia lineatus*	-	NA	-	NA
*Pseudocurimata boehlkei*	-	MH537318.1	-	NA
*Pseudopoecilia fria*	-	NA	-	NA
*Rhamdia cinerascens*	X	MK355304.1	-	NA
*Rhoadsia minor*	X	KY440346.1*	-	NA
*Sicydium salvini*	X	MG496236.1	X	MF927498.1
*Synbranchus marmoratus*	-	NA	-	NA
*Trichomycterus* aff. banneaui	-	NA	-	NA
*Trichomycterus* sp	-	NA	-	NA
*Xiphophorus maculatus*	X	KU692957.1	X	NC_011379.1

“X” denotes that the species match records in the Blastn database between 99–100% of identity, “-” denotes when there is no record with high identity and thus it was not applicable “NA”.

*The close match for *R*. *minor* is *R*. *altipinna*

Based on the tree from the Cube River fish species (Fig 3), our results suggest no taxonomic deviation was detected at the species level, indicating that all examined species could be authenticated by the barcode approach within the species assemblage of the study area. For species with two or more haplotypes, all the haplotypes were associated with their conspecifics in monophyletic clades with high support (S3 and S4 Figs in S1 File).

When analyzing species of the Cube River basin with available sequences (S3 and S4 Figs in S1 File), for some species our identifications did not match available sequences from GenBank or grouped with expected species based on the literature. For example, specimens identified as *Astroblepus* aff. *mindoensis* and *Astroblepus cyclopus* (Astroblepidae: Siluriformes) did not group with Ecuadorian haplotypes like *Astroblepus* cf. *regani*, rather, the former clustered with species from the “Central Andes Clade” [21], and the latter with species from the Magdalena River basin [49] (S3 and S4 Figs in S1 File). COI genetic distance between these samples was 11.3% K2P- and 10.4% p-distance. Because these species have not been sequenced before there was no GenBank match for neither of these species. Similarly, for *Trichomycterus* spp. (Trichomycteridae: Siluriformes), morphologically our specimens are similar *Trichomycterus banneaui* but COI sequence variability suggested that these are genetically different from *T*. *banneaui* of the Magdalena basin, and *Trichomycteru*s sp1 exhibited affinity to other species from different regions in Colombia [22] (S3 Fig in S1 File). COI genetic distance between *Trichomycterus* species were 11.5% K2P- and 10.5% p-distance. In contrast, other siluriforms species like *Pimelodella modestus*, *Chaesthostoma bifurcum* and *Rhamdia guatemalensis*, had concordant morphological classifications and their sequences clustered with known relatives and their own species (S3 Fig in S1 File).

Some Characiformes also showed disagreements between GenBank sequences and Cube River samples. Our COI sequence from *Hoplias microlepis* matched morphological description of this species but showed genetic variation as it diverged earlier than the *H*. *microlepis* sequence from Panama (S3 Fig in S1 File); however, 16S sequence from this species clusters with Panamanian samples of *H*. *microlepis* (S4 Fig in S1 File). The remaining characiforms (*L*. *bimaculata*, *Eretmobrycon festae*, *E*. *cf*. *ecuadoriensis*, *Pseudocurimata boehlkei*, *B*. *atrocaudatus*, *P*. *lineatus* and *Rhoadsia minor*) and species of Cichliformes, Gobiiformes and Cyprinodontiformes, clustered in their respective clades and confirmed their identities by grouping with their previously sequenced homologs since most of these species have been sequenced before.

It is important to note that some species that we have identified in this study differed from previous reported species in the region (Table 1). Although their morphological characters match the descriptions of some species, either their sequence or key diagnostic characters did not match with the formerly proposed species inhabiting the area. Several discrepancies arose including new records, differences in species identification and cryptic diversity. Identified species such as *Andinoacara rivulatus*, *E*. cf. *ecuadoriensis*, *B*. *atrocaudatus*, *H*. *microlepis*, *P*. *modestus*, *P*. *lineatus* and *Trichomycterus* spp, differed in their identification when compared with previous literature from the Cube River (Esmeraldas River basin) (Table 1). In addition, *Sicydium salvini*, *Rhamdia guatemalensis* and *Xiphophorus maculatus* indicate new records for Ecuador overall.

Finally, additional sequence alignments of *Trichomycterus* spp. (n = 8), *Astroblepus* spp. (n = 9) and *P*. *lineatus* (n = 17) exhibited different degrees of variation (S2-S4 Figs in S1 File) with proportions of identical sites of 89.1% (mean diversity: d = 0.06), 90.2% (d = 0.03) and 99.4 (d = 0.00), respectively. *Trichomycterus* spp. had the highest nucleotide diversity. When analyzed together with COI trees and external sequences, we concluded that nucleotide diversity was attributed to cryptic diversity for *Astroblepus* spp. and *Trichomycterus* spp., because after careful examination we were able to discriminate species by a few diagnostic morphological characters (type of adipose fin and spine in *Astroblepus* and type of teeth in *Trichomycterus*).

## 4. Discussion

In this study we have successfully barcoded 26 fish species from the Cube River basin. All targeted species were sequenced and identified to the lowest possible taxonomic level using molecular and morphological tools. Most fish species identities matched previous species known to inhabit this region while others suggested misidentifications or new records when compared from previous studies. A few species represent new records in the area and several species were sequenced for the first time. Our study suggests that DNA barcoding coupled with morphological considerations and literature reviews are a useful approach in classifying species, and that it is a suitable exploratory tool to understand diversity in the Ecuadorian CGE.

### 4.1 Barcoding

DNA barcoding for identifying fish species has been gaining attention for decades and with different applications that range from characterizing fish diversity inhabiting river systems [50], island freshwater systems [25] or marine ecosystems [23], to efforts in correctly identifying species from artisanal fisheries markets [51, 52] and illegal fishing vessels [53]. While DNA barcoding has proven to be a valuable tool for fish identification, there are potential challenges associated with its use. The success of fish barcoding studies could be limited by introgression and hybridization [54], high intraspecific variation [55], incomplete reference databases [56], and quality of DNA [57].

Our study aligns itself in barcoding efforts by improving our understanding of Ecuadorian species. Many CGE species are endemic and endangered [15], and some were sequenced for the first time, contributing to the current and future knowledge and conservation value of freshwater fishes in Ecuador. Although COI is the most used gene in barcoding for freshwater fish, we also used 16S rRNA as a complementary gene marker in case COI-sequences of analyzed species were absent in GenBank or if species were not successfully barcoded with COI marker. This was useful in our case for *G*. *maculatus* because we could not barcode this species with COI (Table 2). This study produced 16S sequences, which will now be available to be used by researchers in future phylogenetic studies using several gene markers.

Further, in our sequence library comprising 26 fish species, all sequenced haplotypes of the same species formed high bootstrap-supported clusters in the COI-16s based tree (Fig 3). The high discrimination power of DNA barcoding in our data set occurred because most genera were represented by only one species (26 species from 23 genera); therefore, the number of closely related congeners was quite low. Although our data set is valuable and will help as an identification tool for fish research from the Ecuadorian CGE and the Esmeraldas River basin, careful consideration should be taken when integrating this information in fish identification. The exclusive use of mitochondrial DNA does not eliminate the risk of having introgression or hybridization cases between closely related species (e.g., the case of *Rhoadsia* [28]), which could hinder accurate species identification using only mitochondrial barcodes. Studies should also incorporate nuclear genetic information to aid in species discrimination, along with other lines of evidence such as morphological measurements and ecological interactions.

### 4.2 The need for further research

The mismatches found in our study between species identities and previous records in the literature (Table 1) coupled with the phylogenetic variability of our analyzed species with the same taxa from different regions (e.g. *H*. *micolepis*, *R*. *guatemalensis*) highlights the incomplete knowledge of the CGE ichthyofauna, and the apparent discrepancy between present and historical occurrence data, which could be attributed to extinction, synonymy, misidentifications and new records.

Some of these differences could be easily resolved by analyzing the respective updated systematics of each group for future species lists in the region. For example, the species status of the cichlid *Andinoacara blombergi*, which has been questioned after research of Neotropical cichlids showed no genetic differences between *A*. *blombergi* and *A*. *rivulatus*, with *A*. *rivulatus* being the main lineage of western Ecuador [37], should be updated. Similarly, the validity of *Rhamdia quelen* in trans-Andean basins has been challenged, and *Rhamdia cinerascens* is now the current lineage for Western Ecuador [58]. However, the presence of *R*. *cinerascens* is based on specimens from the southern Daule River, Guayas River basin [47, 59]; more than 200 km apart from the Esmeraldas River basin. Our morphological and genetic analyses suggest the species from the Cube River is *Rhamdia guatemalensis*, which has a western-Colombian distribution, including the southern region [46]; thus, we recommend the addition of this species to the CGE list of Ecuadorian fishes. Finally, our sample from *Sicydium*, based on COI and 16S sequences and morphological characteristics such as tricuspid teeth suggest complete match with the species *Sicydium salvini*, yet only S. *hildebrandii and S*. *rosenbergii* have been reported for the Ecuadorian CGE and they have not been sequenced. This record represents a southern expansion for *S*. *salvini* [8, 59] and that a review of the species should be considered, given the sparse occurrence of *Sicydium* species in sampling efforts [60]. It is important to note that S. *salvini* was mentioned to occur in Ecuador in the 2012 fish list [61] but it was deleted from subsequent lists.

Our results suggest the presence of more than one species for some specific groups that are taxonomically complex and that are known to have several unidentified lineages in South America [21, 22]. For instance, *Astroblepus* samples clustered variably within species from Colombia and Pánama (S3 and S4 Figs in S1 File), suggesting that the two species from this study belong to different clades. Upon sequencing additional samples, we identified one specimen from site 10 as belonging to the species *Astroblepus cyclopus*. The occurrence of two *Astroblepus* species in the Cube River basin was supported by genetic distance, which showed at least a 10% divergence with the other *Astroblepus* species in the basin. In *Astroblepus* studies, different lineages have been identified using a threshold of 1% divergence [21]. This finding was also corroborated by morphological measurements (see Results section) and by the known distribution of *A*. *cyclopus* in Andean River networks and in the Esmeraldas River basin [62]. The systematics of *Astroblepus* in Ecuador is not completely understood and regional efforts still lack Ecuadorian samples [21]. For *Trichomycterus*, our samples cluster with Colombian species but species from Ecuador are still absent from large-scale studies [63]. *Trichomycterus* specimens did show genetic variability (S5 Fig in S1 File), where the ones used in the tree exhibited genetic divergence and morphological examination corroborated this by separating them in two sympatric species: *T*. aff *banneaui* and *Trichomycterus* sp1. It is important to note that *Trichomycterus* populations may have high fidelity to their headwater habitats and may remain isolated throughout the hydrological cycle of the Cube River basin; *Trichomycterus* were the only species found in isolated pools in the dry season. The species *A*. aff *mindoensis*, *T*. aff. *banneaui* and *Trichomycterus* sp. 1, are probably new to science and should be further studied. Additionally, because *Hoplias microlepis* from Ecuador places separately from *H*. *microlepis* (samples from Panama) and *H*. *malabaricus* (samples from Brazil) in the trees (S3 and S4 Figs in S1 File), this highlights the need to perform deeper genetic and morphological studies with the Ecuadorian species from different western basins. This is particularly necessary for *Hoplias* species given the known species complex of *Hoplias malabaricus* harboring several subclades (among them *H*. *microlepis*) [20, 64], the disjoint distribution of *H*. *microlepis* between Pánama and Ecuador [47] and the proclivity of *Hoplias microlepis* to quickly accumulate genetic differences between populations [29].

Finally, we detected introduced species through barcoding. Tilapia (*Oreochromis niloticus*) and the guppy (*Poecilia reticulata*) have already been reported to be present in western Ecuador in the Atacames River and in the Guayas River basins [17, 61]. Here we report these species for the first time in the Esmeraldas River basin. The invasive platy, *Xiphophorus maculatus* is reported for the first time in Ecuador. The expansion in its distribution range to Ecuador could be related to natural migration from other invasive populations that have been detected in the Magdalena River in Colombia [65], but since *X*. *maculatus* was found in a single river (site 13) that is affluent from the Cube Lagoon near the Mache Chindul reserve, where platys have been previously observed (personal observation), this was most likely product of single human-based introduction event in the Lagoon.

Greater knowledge of fish diversity in this threatened region [1], that exhibits intermittency [5] and makes it more vulnerable to climate change, will benefit the local government and stakeholders, to develop conservation measures. Filling knowledge gaps of fish diversity could improve long-term conservation plans of aquatic ecosystems, particularly for the conservation of specific species for which information was previously unavailable. Our results highlight the importance of preserving streams in the upper part of the river network. In these areas, specialized and phylogenetically unique species are found, and no introduced species occur. Further, there are localities that contribute to biodiversity because species from these sites did not occur anywhere else, highlighting the need to study and preserve these specific locations.

## Supporting information

S1 File Supplementary material. (DOCX)
