# Supplementary material for: New records of native and introduced fish species in a river basin of Western Ecuador, the Chocó-Darien Ecoregion, using DNA barcoding
Source: PLoS One. 2024 Mar 8;19(3):e0298970. doi: 10.1371/journal.pone.0298970 (PMC10923491; doi:10.1371/journal.pone.0298970)
Supplement: S1 File — (DOCX) [file pone.0298970.s001.docx]

**Supplementary material**

**S1 Table.** Sampling sites in this study.

| **Sampling site** | **Latitude** | **Longitude** | **Altitude (m)** |
| --- | --- | --- | --- |
| CUB 01 | 0°22'40.980"N | 79°39'52.5600"W | 507 |
| CUB 02 | 0°21'54.9720"N | 79°42'05.9760"W | 342 |
| CUB 03 | 0°22'07.9680"N | 79°40'07.6800"W | 472 |
| CUB 04 | 0°21'03.6000"N | 79°40'58.8000"W | 518 |
| CUB 05 | 0°21'06.9120"N | 79°40'51.7080"W | 512 |
| CUB 06 | 0°21'04.4640"N | 79°40'41.1240"W | 464 |
| CUB 07 | 0°21'11.7828"N | 79°40'39.6300"W | 531 |
| CUB 08 | 0°22'31.9800"N | 79°39'27.0000"W | 332 |
| CUB 09 | 0°23'21.3360"N | 79°40'24.6000"W | 351 |
| CUB 10 | 0°22'05.9880"N | 79°41'08.9880"W | 526 |
| CUB 11 | 0°24'43.9920"N | 79°40'03.0000"W | 198 |
| CUB 12 | 0°25'22.8000"N | 79°41'34.8000"W | 376 |
| CUB 13 | 0°24'50.4000"N | 79°38'56.4000"W | 215 |
| CUB 14 | 0°26'42.9720"N | 79°39'04.9680"W | 133 |
| CUB 15 | 0°26'37.9680"N | 79°39'04.9680"W | 135 |
| CUB 16 | 0°25'30.0072"N | 79°37'21.3420"W | 207 |
| CUB 17 | 0°25'08.4000"N | 79°39'00.0000"W | 208 |
| CUB 18 | 0°30'50.4000"N | 79°38'56.4000"W | 86 |
| CUB 19 | 0°31'17.0004"N | 79°38'58.2000"W | 78 |
| CUB 20 | 0°33'45.9720"N | 79°38'01.9680"W | 52 |

**S2 Table.** Genes and primers used in this study.

| Genes and Primers | Sequence (5`-3`) | Source |
| --- | --- | --- |
| Mitochondrial 16S |  |  |
| 16SaL-F | ACGCCTGTTTATCAAAAACAT | [40] |
| 16Sbr-R | CCGGTCTGAACTCAGATCACGT | [40] |
| Mitochondrial COI |  |  |
| FISH-BCL | TCAACYAATCAYAAAGATATYGGCAC | [41] |
| FISH-BCH | TAAACTTCAGGGTGACCAAAAAATCA | [41] |

**S3 Table.** Thermocycling conditions used to amplify mitochondrial genes using polymerase chain reaction (PCR).

| Genes | Protocol |
| --- | --- |
| 16S | 1 cycle: 5 min 94°C |
|  | 35 cycles: 45 s 94°C, 45 s 53°C, 60 s 72°C |
|  | 1 cycle: 7 min 72°C |
| COI | 1 cycle: 5 min 95°C |
|  | 35 cycles: 30 s 95°C, 30 s 52°C, 45 s 72°C |
|  | 1 cycle: 5 min 72°C |

*Protocols were developed by [40] and [41], respectively.

**S4 Table.** GenBank sequences used in this study. Accession numbers in bold denote sequences generated in this study.

| **Taxa** | **16S** | **Taxa** | **COI** |
| --- | --- | --- | --- |
|  | Accession # |  | Accession # |
| *Agonostomus monticola* | JQ060645.1 | *Agonostomus monticola* | MG936878.1 |
| *Agonostomus monticola* | JQ060648.1 | *Agonostomus monticola* | **OR395359** |
| *Agonostomus monticola* | KF375044.1 | *Agonostomus monticola* | **OR395326** |
| *Agonostomus monticola* | **OR395154** | *Andinoacara coeruleopunctatus* | MG936664.1 |
| *Andinoacara rivulatus* | EF432885.1 | *Andinoacara rivulatus* | KU692245.1 |
| *Andinoacara rivulatus* | EF432886.1 | *Andinoacara rivulatus* | **OR395329** |
| *Andinoacara rivulatus* | Mt508813.1 | *Andinoacara rivulatus* | MH444791.1 |
| *Andinoacara rivulatus* | **OR395162** | *Arapaima gigas* | MH830243.1 |
| *Andinoacara rivulatus* | **OR395163** | *Astroblepus* cf. *regani* | MN542105.1 |
| *Astroblepus* aff. *trifasciatus* | MN541468.1 | *Astroblepus* cf. *regani* | MN542106.1 |
| *Arapaima gigas* | AY504834.1 | Astroblepus gr. *grixalvii* | MN542047.1 |
| *Astroblepus* cf *regani* | MN541469.1 | *Astroblepus* gr. *grixalvii* | MN542046.1 |
| *Astroblepus* sp. 2 | KP959743.1 | *Astroblepus* sp. | MN542143.1 |
| *Astroblepus* sp. 18 | MN541416. | *Astroblepus* sp. | MN542115.1 |
| *Astroblepus* sp. 19 | MN541418.1 | *Astroblepus* sp. | HM049074.1 |
| *Astroblepus* sp. 25 | MN541474.1 | *Astroblepus* sp. | MG936675.1 |
| *Astroblepus* sp. | HM049029.1 | *Astroblepus cyclopus* | **OR395334** |
| *Astroblepus aff. mindoensis* | **OR395126** | *Astroblepus* aff. mindoensis | **OR395335-42** |
| *Astroblepus cyclopus* | **OR395127** | *Astyanax festae* | KY268284.1 |
| *Awaous banana* | KF415310.1 | *Astyanax ruberrimus* | MG936706.1 |
| *Awaous banana* | MF927489.1 | *Awaous banana* | MG936713.1 |
| *Awaous grammepomus* | MH699835.1 | *Awaous banana* | MG936712.1 |
| *Awaous transandeanus* | **OR395156** | *Awaous transandeanus* | MZ130160.1 |
| *Awaous transandeanus* | **OR395157** | *Awaous transandeanus* | MZ130161.1 |
| *Awaous transandeanus* | **OR395158** | *Awaous transandeanus* | **OR395323** |
| *Brachyplatystoma juruense* | KR260097.1 | *Awaous transandeanus* | **OR395324** |
| *Brycon henni* | FJ944720.1 | *Awaous transandeanus* | **OR395325** |
| *Brycon* sp. | OP422488.1 | *Brycon argenteus* | MG936800.1 |
| *Brycon atrocaudatus* | **OR395140** | *Brycon atrocaudatus* | **OR395327** |
| *Brycon atrocaudatus* | **OR395141** | *Brycon atrocaudatus* | **OR395328** |
| *Eretmobrycon dahli* | KF209727.1 | *Brycon chagrensis* | MG936804.1 |
| *Eretmobrycon emperador* | HQ171266.1 | *Brycon petrosus* | MG936811.1 |
| *Carlana eigenmanni* | OP647421.1 | *Brycon* sp | OP418233.1 |
| *Chaetostoma bifurcum* | OL303483.1 | *Brycon* sp | OP418235.1 |
| *Chaetostoma bifurcum* | **OR392418** | *Carlana eigenmanni* | MZ820764.1 |
| *Chaetostoma bifurcum* | **OR395128** | *Chaetostoma bifurcum* | **OR395333** |
| *Chaetostoma fischeri* | OL303524.1 | *Chaetostoma fischeri* | MG936833.1 |
| *Chaetostoma sp.* | KP959851.1 | *Eretmobrycon brevirostris* | KF210042.1 |
| *Dormitator latifrons* | KX095211.1 | *Eretmobrycon brevirostris* | MH003099.1 |
| *Eretmobrycon brevirostris* | KF209716.1 | *Eretmobrycon dahli* | KF210055.1 |
| *Eretmobrycon emperador* | KF209733.1 | *Eretmobrycon ecuadoriensis* | **OR395320** |
| *Eretmobrycon peruanus* | KF209754.1 | *Eretmobrycon emperador* | MG936792.1 |
| *Eretmobrycon festae* | **OR395142** | *Eretmobrycon festae* | **OR395321** |
| *Eretmobrycon festae* | **OR395143** | *Eretmobrycon peruanus* | KF210072.1 |
| *Eretmobrycon ecuadoriensis* | **OR395144** | *Hoplias malabaricus* | HM906018.1 |
| *Eretmobrycon ecuadoriensis* | **OR395145** | *Hoplias malabaricus* | MG699489.1 |
| *Gobiomurus maculatus* | KX095212.1 | *Hoplias malabaricus* | FBCH094-21 |
| *Gobiomurus maculatus* | KF415378.1 | *Hoplias malabaricus* | MG936989.1 |
| *Gobiomorus maculatus* | **OR395152** | *Hoplias microlepis* | MG936994.1 |
| *Gobiomorus maculatus* | **OR395153** | *Hoplias microlepis* | MG936997.1 |
| *Goeldiella eques* | JF898719.1 | *Hoplias microlepis* | MG937000.1 |
| *Hoplias malabaricus* | HQ171346.1 | *Hoplias microlepis* | **OR395348** |
| *Hoplias microlepis* | MT372726.1 | *Lebiasina festae* | MG937035.1 |
| *Hoplias microlepis* | HQ171264.1 | *Lebiasina bimaculata* | **OR395343** |
| *Hoplias* sp. | AY788031.1 | *Lebiasina bimaculata* | **OR395344** |
| *Hoplias microlepis* | **OR395131** | *Lebiasina panamensis* | MG937042.1 |
| *Joturus pichardi* | JF911708.1 | *Mesoheros atromaculatus* | MG937052.1 |
| *Lebiasina astrigata* | AP011995.1 | *Mesoheros festae* | DQ119216.1 |
| *Lebiasina multifasciata* | AP006766.1 | *Mesoheros festae* | **OR395330** |
| *Lebiasina bimaculata* | **OR395132** | *Mesoheros festae* | **OR395331** |
| *Lebiasina bimaculata* | **OR395133** | *Oreochromis aureus* | MK130704.1 |
| *Lebiasina bimaculata* | **OR395134** | *Oreochromis niloticus* | **OR395332** |
| *Mesoheros festae* | DQ119187.1 | *Oreochromis niloticus* | KU565830.1 |
| *Mesoheros festae* | **OR395159** | *Osteoglossum bicirrhosum* | KU568947.1 |
| *Mesoheros festae* | **OR395160** | *Parastremma sadina* | MZ820762.1 |
| *Ophisternon aenigmaticum* | KC880333.1 | *Pimelodella modesta* | MW830110.1 |
| *Oreochromis mossambicus* | MH767419.1 | *Pimelodella* sp. | MG937112.1 |
| *Oreochromis niloticus* | MH567058.1 | *Pimelodella modestus* | **OR395346** |
| *Oreochromis niloticus* | **OR395161** | *Pimelodella modestus* | **OR395347** |
| *Osteoglossum bicirrhosum* | AY504830.1 | *Poecilia reticulata* | JN028265.1 |
| *Parastremma sadina* | OP647422.1 | *Poecilia reticulata* | **OR395294** |
| *Pimelodella cristata* | MH286809.1 | *Poecilia reticulata* | **OR395295** |
| *Pimelodella modestus* | **OR395124** | *Priapichthys darienensis* | MG937149.1 |
| *Pimelodella modestus* | **OR395125** | *Pseudochalceus kyburzi* | HM562869.1 |
| *Poecilia reticulata* | KX816038.1 | *Pseudochalceus lineatus* | **OR395303-19** |
| *Poecilia reticulata* | OP863306.1 | *Pseudocurimata boehlkei* | MH537319.1 |
| *Poecilia reticulata* | **OR395148** | *Pseudocurimata boehlkei* | **OR395298** |
| *Poecilia reticulata* | **OR395149** | *Pseudocurimata boehlkei* | **OR395299** |
| *Pseudochalceus kyburzi* | HM562765.1 | *Pseudocurimata boulengeri* | MH537320.1 |
| *Pseudochalceus lineatus* | **OR395136** | *Pseudocurimata lineopunctata* | MH537322.1 |
| *Pseudochalceus lineatus* | **OR395137** | *Pseudocurimata troschelii* | MH537326.1 |
| *Pseudocurimata boehlkei* | **OR395138** | *Pseudopoecilia fria* | **OR395356** |
| *Pseudocurimata boehlkei* | **OR395139** | *Rhamdia cinerascens* | MT469159.1 |
| *Pyrrhulina australis* | HQ171293.1 | *Rhamdia cinerascens* | MT469160.1 |
| *Pseudopoecilia fria* | **OR395150** | *Rhamdia guatemalensis* | MT469161.1 |
| *Pseudopoecilia fria* | **OR395151** | *Rhamdia guatemalensis* | MW476063.1 |
| *Rhamdia guatemalensis* | **OR395123** | *Rhamdia guatemalensis* | **OR395360** |
| *Rhoadsia minor* | OP647424.1 | *Rhamdia guatemalensis* | **OR395345** |
| *Rhoadsia minor* | **OR395135** | *Rhamdia quelen* | MG937188.1 |
| *Sicydium altum* | MF927496.1 | *Rhamdia* sp | MK355304.1 |
| *Sicydium plumieri* | HQ639104.1 | *Rhoadsia altipinna* | KU252647.1 |
| *Sicydium salvini* | KF415466.1 | *Rhoadsia minor* | KY440351.1 |
| *Sicydium salvini* | MF927498.1 | *Rhoadsia minor* | **OR395300** |
| *Sicydium* sp | KF415467.1 | *Rhoadsia minor* | **OR395301** |
| *Sicydium salvini* | **OR395155** | *Rhoadsia minor* | **OR395302** |
| *Steindachnerina guentheri* | FJ944748.1 | *Rhoadsia minor* | MZ820736.1 |
| *Steindachnerina insculpta* | HQ171339.1 | *Roeboides occidentalis* | MG937248.1 |
| *Synbranchus marmoratus* | KC880317.1 | *Sicydium salvini* | MK655581.1 |
| *Synbranchus* sp. | ON401040.1 | *Sicydium salvini* | MG496237.1 |
| *Synbranchus marmoratus* | **OR395122** | *Sicydium salvini* | MG937264.1 |
| *Trichomuyterus areolatus* | AP012026.1 | *Sicydium salvini* | MG496236.1 |
| *Trichomycterus barbourin* | MW415496.1 | *Sicydium salvini* | **OR395322** |
| *Trichomycterus bogotense* | MW415497.1 | *Synbranchus marmoratus* | MG937294.1 |
| *Trichomycterus chapmani* | MW415498.1 | *Synbranchus marmoratus* | MG937288.1 |
| *Trichomycterus conradi* | MT025530.1 | *Synbranchus marmoratus* | MG937280.1 |
| *Trichomycterus retropinnis* | MW415507.1 | *Synbranchus marmoratus* | **OR395357** |
| *Trichomycterus aff. banneaui* | **OR395129** | *Trichomycterus banneaui* | KY858001.1 |
| *Trichomycterus sp1* | **OR395130** | *Trichomycterus guacamayoensis* | MH407227.1 |
| *Xiphophorus helleri* | FJ234985.1 | *Trichomycterus* sp | MK355341.1 |
| *Xiphophorus maculatus* | NC_011379. | *Trichomycterus* aff. *banneau*i | **OR395358** |
| *Xiphophorus maculatus* | **OR395146** | *Trichomycterus* aff. *banneau*i | **OR395349** |
| *Xiphophorus maculatus* | **OR395147** | *Trichomycterus* aff. *banneau*i | **OR395350** |
|  |  | *Trichomycterus* aff. *banneau*i | **OR395351** |
|  |  | *Trichomycterus* aff. *banneau*i | **OR395352** |
|  |  | *Trichomycterus* sp1 | **OR395353** |
|  |  | *Trichomycterus* sp1 | **OR395354** |
|  |  | *Trichomycterus* sp1 | **OR395355** |
|  |  | *Xiphophorus maculatus* | KU692957.1 |
|  |  | *Xiphophorus maculatus* | **OR395296** |
|  |  | *Xiphophorus maculatus* | **OR395297** |

**S5 Table.** Evolution models selected according to Best-fit model by Bayesian Information Criterion.

| Alignment | Model |
| --- | --- |
| Part 1 (16S-concatenated) | TIM2+F+I+G4 |
| Part 2 (COI-concatenated) | TPM3+F+I+G4 |
| COI | TVM+F+R5 |
| 16S | TIM2+F+I+G4 |

**S6 Table.** Diagnostic morphological characters used to identify species of the Ecuadorian Chocó-Darien Global Ecoregion.

| **Taxa** | **Characters from the Ecuadorian CGE** | **Ref.** |
| --- | --- | --- |
| CICHLIFORMES |  |  |
| CICHLIDAE |  |  |
| *Andinoacara rivulatus* | 3 anal spines | [1][2] |
|  | 17-20 scales in upper lateral line (LL) |  |
|  | 5 dark vertical bars |  |
|  | Head width 16.65-20.81% in standard length (SL) |  |
|  | Preorbital length 11.38-17.74 in SL |  |
| *Mesoheros festae* | 4 anal spines | [1] |
|  | 23 scales in upper LL |  |
|  | 9 dark Vertical bars |  |
|  | Presence of a dark spot in the upper base of caudal fin |  |
| CYPRINODONTIFORMES |  |  |
| POECILIIDAE |  |  |
| *Xiphophorus maculatus* | 8-9 dorsal fin rays | [3] |
|  | Gonopodium length greater than head |  |
|  | 25-26 lateral line scales |  |
| *Pseudopoecilia fria* | 28-30 LL scales | [1] |
|  | Curved gonopodium |  |
|  | Dorsal fin origin behind anal fin |  |
| CHARACIFORMES |  |  |
| CURIMATIDAE |  |  |
| Pseudocurimata boehlkei | 45 LL scales | [1] |
|  | 6 scales from dorsal fin to LL |  |
|  | 5 scales from LL to anal fin |  |
|  | Horizontal peduncle elongated blotch |  |
| CHARACIDAE |  |  |
| *Eretmobrycon festae* | Presence of two humeral dark spots | [1][4] |
|  | 4 teeth on second premaxillary row |  |
|  | No maxillary teeth |  |
| *Eretmobrycon ecuadoriensis* | Humeral spot absent (or inconspicuous) or present (conspicuous) | [5] |
|  | 34-39 LL scales |  |
|  | 24-27 anal rays |  |
|  | Presence of a caudal spot in the peduncle |  |
|  | Elongated caudal spot |  |
| *Rhoadsia minor* | Body height 1.6-3.4 times in SL | [6][7] |
| *Pseudochalceus lineatus* | 26 anal rays | [1] |
|  | Presence of Longitudinal stripes |  |
| BRYCONIDAE |  |  |
| *Brycon atrocaudatus* | Upper jaw surpasses lower jaw leaving teeth expose only from the first row | [1] |
|  | Snout 3 to 3, 5 times length in SL |  |
|  | 17-24 anal rays |  |
|  | 49-55 LL scales |  |
|  | Pectoral fin does not reach origin of pelvic fin |  |
| LEBIASINIDAE |  |  |
| *Lebiasina bimaculata* | Body height 3.9-4.7 times in in SL | [1] |
|  | Presence of black spot in the caudal base |  |
|  | Presence of humeral spot behind the head |  |
|  | Presence of humeral red dot |  |
| ERYTHRINIDAE |  |  |
| *Hoplias* aff *microlepis* | 40-45 LL scales | [8] |
|  | 10-11 rows of scales around caudal peduncle |  |
| GOBIIFORMES |  |  |
| GOBIIDAE |  |  |
| *Gobiomurus maculatus* | Big gill opening | [1] |
|  | Presence of conical teeth in upper jaw |  |
|  | Preopercle with spine |  |
|  | Presence of a toothed vomer |  |
| *Sicydium salvini* | Tricuspid teeth | [9][10] |
|  | Smooth surface of upper lip |  |
|  | Jaw extent beyond middle of the eye |  |
| *Awaous transandeanus* | 5-6 pores per opercle, preopercle and eye | [1] |
|  | Lower jaw with multiple teeth rows |  |
| SILURIFORMES |  |  |
| TRICHOMYCTERIDAE |  |  |
| *Trychomycterus* sp1 | Type of teeth incisive | [1] |
|  | Caudal fin shape truncated or rounded |  |
|  | Origin of anal fin at half dorsal fin or behind dorsal fin |  |
|  | Spots in irregular patterns not forming rows |  |
| *Trychomycterus* aff. banneaui | Type of teeth conical or incisive | [1] |
|  | Caudal fin shape truncated or rounded |  |
|  | Origin of anal fin at half dorsal fin or behind dorsal fin |  |
|  | Spots in the middle forming longitudinal rows |  |
| ASTROBLEPIDAE |  |  |
| *Astroblepus* aff. *mindoensis* | Adipose fin with embedded small spine. | [1] |
|  | Teeth of the first row of the premaxilla unicuspid, a few at the center bicuspid |  |
|  | Developed fleshy adipose fin |  |
| *Astroblepus* cyclopus | Adipose fin with free large spine |  |
|  | Teeth of the first row of the premaxilla unicuspid, a few at the center bicuspid |  |
|  | Undeveloped adipose fin |  |
| HEPTATERIDAE |  |  |
| *Rhamdia* aff *guatemalenssis* | Length of dorsal fin base in SL | [11][12] |
|  | Length of adipose fin base in SL |  |
|  | Adipose fin equidistant to dorsal and caudal fin |  |
|  | Outer barberls reach base of pectoral fin rays |  |
|  | Extended barbels beyond pelvic fins |  |
|  | Head length/SL proportion |  |
|  | Lateral line conspicuous or not conspicuous |  |
| *Pimelodella* aff. *modestus* | Adipose fin short or long (<3.5 or 4 to 5 times) | [1] |
|  | Barbels reach posterior part of ventral fins |  |
|  | Serrated hooks on pectoral fin big or small |  |
|  | Size of caudal fin lobes |  |
| LORICARIIDAE |  |  |
| *Chaetostoma bifurcum* | 2 Cheek odontodes | [13] |
|  | 4 anal fin rays |  |
|  | 8 dorsal fin rays |  |
| MUGILIFORMES |  |  |
| MUGILIDAE |  |  |
| *Agonostomus monticola* | Interorbital space convex | [1] |
|  | 2 spines on anal fin |  |
|  | Dorsal color olive grey |  |

[1]: (Jiménez-Prado et al., 2015); [2] (Wijkmark et al., 2012): [3] (Albornoz-Garzón and Villa-Navarro, 2017), [4] (Terán et al., 2020), [5] (Román–Valencia et al., 2015), [6] (Cucalón et al., 2022), [7] (Malato et al., 2017), [8] (Mattox et al., 2014), [9] (Bussing, 1996), [10] (Chabarria and Pezold, 2013), [11] (Hernández et al., 2015), [12] (Perdices et al., 2002), [13] (Lujan et al., 2015)

**S7 Table.** Species found in this study and their distribution in coastal Ecuador. SC = Santiago Cayapas drainage system, E = Esmeraldas River basin, NC = Northern coastal area (drainage systems between Esmeraldas and Guayas basins), G = Guayas River basin, SoC = Southern coastal area (river networks of the Catamayo zone).

| Order | Family | Species | Distribution | Ref. |
| --- | --- | --- | --- | --- |
| Mugiliformes | Mugilidae | *Agonostomus monticola* | SC, E, NC, G, SoC | [1][2] |
| Cichliformes | Cichlidae | *Andinoacara rivulatus* | E, G, SoC | [1] |
| Siluriformes | Astroblepidae | *Astroblepus aff mindoensis** | E | [3] |
| Siluriformes | Astroblepidae | *Astroblepus cyclopus* | E, SC | [1][4] |
| Gobiiformes | Gobiidae | *Awaous transandeanus* | SC, E, NC, G, SoC | [1][5] |
| Characiformes | Bryconidae | *Brycon atrocaudatus** | E, G, SoC | [1][6] |
| Characiformes | Characidae | *Eretmobrycon festae* | E, G, SoC | [1] |
| Characiformes | Characidae | *Eretmobrycon ecuadoriensis** | SC, E, NC, G, SoC | [1] |
| Siluriformes | Loricariidae | *Chaethostoma bifurcum** | E, G, SoC | [1] |
| Gobiiformes | Eleotridae | *Gobiomorus maculatus* | SC, E, NC, G, SoC | [1] |
| Characiformes | Erythrinidae | *Hoplias microlepis* | E, G, SoC | [7][3] |
| Characiformes | Lebiasinidae | *Lebiasina bimaculata* | SC, E, NC, G, SoC | [1] |
| Cichliformes | Cichlidae | *Mesoheros festae* | E, NC, G, SoC | [1] |
| Cichliformes | Cichlidae | *Oreochormis niloticus*^†^ | E, NC, G | [1][3][8] |
| Siluriformes | Heptateridae | *Pimelodella aff. modestus* | E, NC, G, SoC | [1] |
| Cyprinodontiformes | Poeciliidae | *Poecilia reticulata*^†^ | SC, E, NC, G | [1][3] |
| Characiformes | Characidae | *Pseudochalceus lineatus** | SC, E, NC, G | [1] |
| Characiformes | Curimatidae | *Pseudocurimata boehlkei** | SC, E | [1] |
| Cyprinodontiformes | Poeciliidae | *Pseudopoecilia fria** | SC, E, NC, G, SoC | [1] |
| Siluriformes | Heptateridae | *Rhamdia guatemalensis* | E | [3] |
| Characiformes | Characidae | *Rhoadsia minor** | SC, E | [1] |
| Gobiiformes | Gobiidae | *Sicydium salvini* | E | [3] |
| Synbranchiformes | Synbranchiidae | *Synbranchus marmoratus* | SC, E, NC, G, SoC | [1] |
| Siluriformes | Trichomycteridae | *Trichomycterus* aff. *banneaui** | E | [3] |
| Siluriformes | Trichomycteridae | *Trichomycterus* sp1* | E | [3] |
| Cyprinodontiformes | Poeciliidae | *Xiphophorus maculatus*^†^ | E | [3] |

References: [1] (Navarrete-Amaya et al, 2021), [2] (NatureServe and Lyons, 2019) [3] This study, [4] (Arguello et al., 2016), [5] (McMahan and Elias, 2020), [6] (Lyons, 2021), [7](Lyons, 2020), [8] (Jiménez-Prado P, 2021). ***** = endemic, **^†^** = introduced, the remaining species are native to the Neotropics.

**S8 Table**. Nucleotide statistics of COI and 16S alignments

|  | COI (n=40) | 16S (n=43) |
| --- | --- | --- |
| Avg. length | 634.4 | 568.7 |
| Min. | 466 | 490 |
| Max. | 667 | 604 |
| Std Dev: | 46.8 | 29.3 |
| Identical sites | 55.6 % | 51.1% |
| Identity | 79.9 % | 81.6 % |
| T | 24.4 % | 22.5 % |
| C | 18 % | 24.7 % |
| A | 30.5 % | 30.7 % |
| G | 27 % | 22.2 % |
| GC | 45.1 | 46.9 % |
| # Sequences | 39 | 42 |
| d* | 0.20 | 0.16 |

*Mean evolutionary diversity for the entire population

**S9 Table.** Genetic divergence (percentage, K2P and p-distance distance) within each gene dataset.

|  | **COI** | **16S** |
| --- | --- | --- |
| **K2P** |  |  |
| Mean | 24 | 19.03 |
| Min. | 0.00 | 0.00 |
| Max. | 32.38 | 30.48 |
| **P-distance** |  |  |
| Mean | 20.05 | 16.48 |
| Min. | 0.00 | 0.00 |
| Max. | 25.96 | 24.8 |


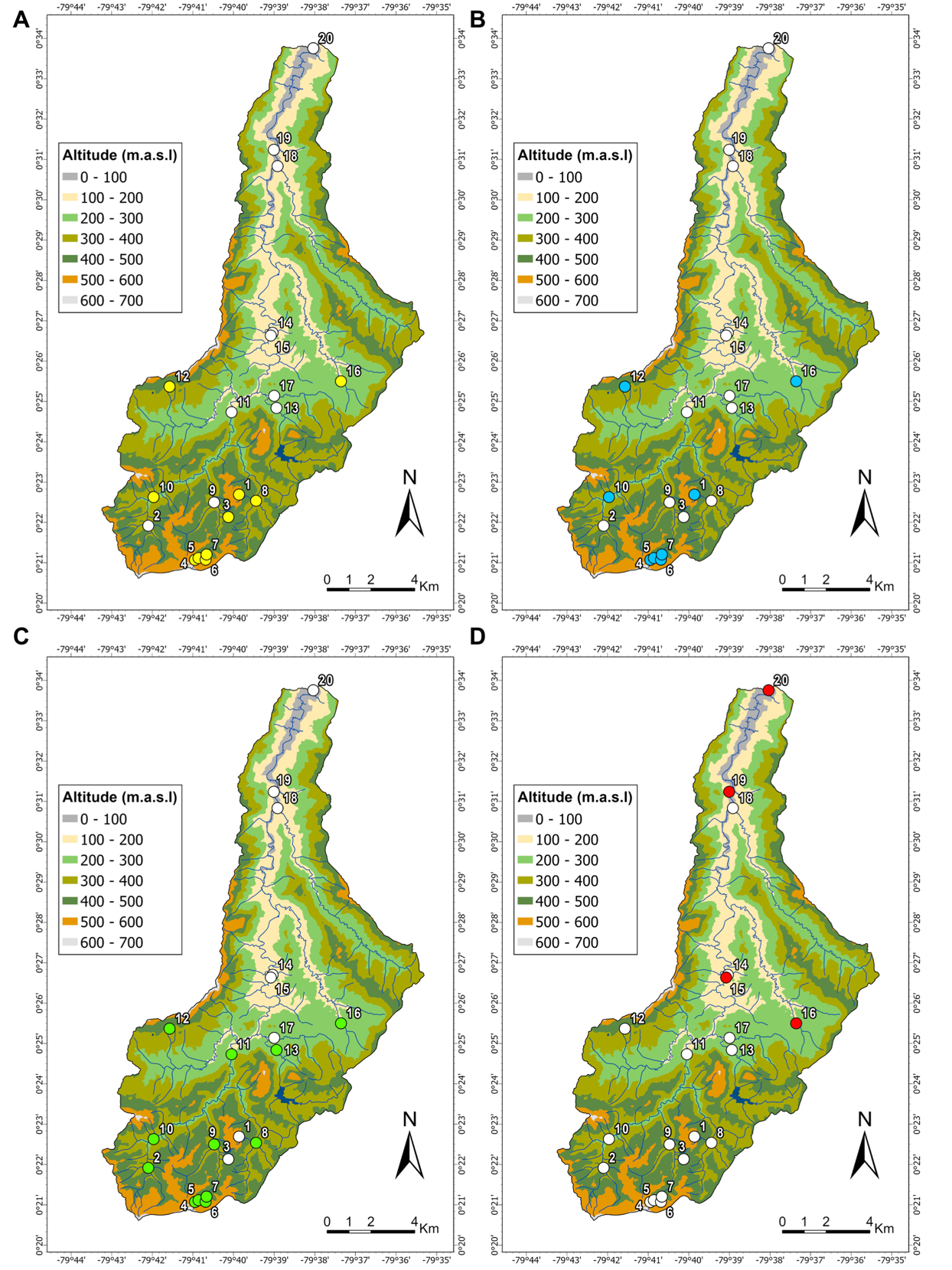


**Figure S1:** Distribution of A) *Trichomycterus* spp., B) *Astroblepus* spp., C) *Pseudochalceus lineatus* and D) amphidromous species in the Cube River basin and associated headwaters. Colored circles indicate the sites where each species were found.


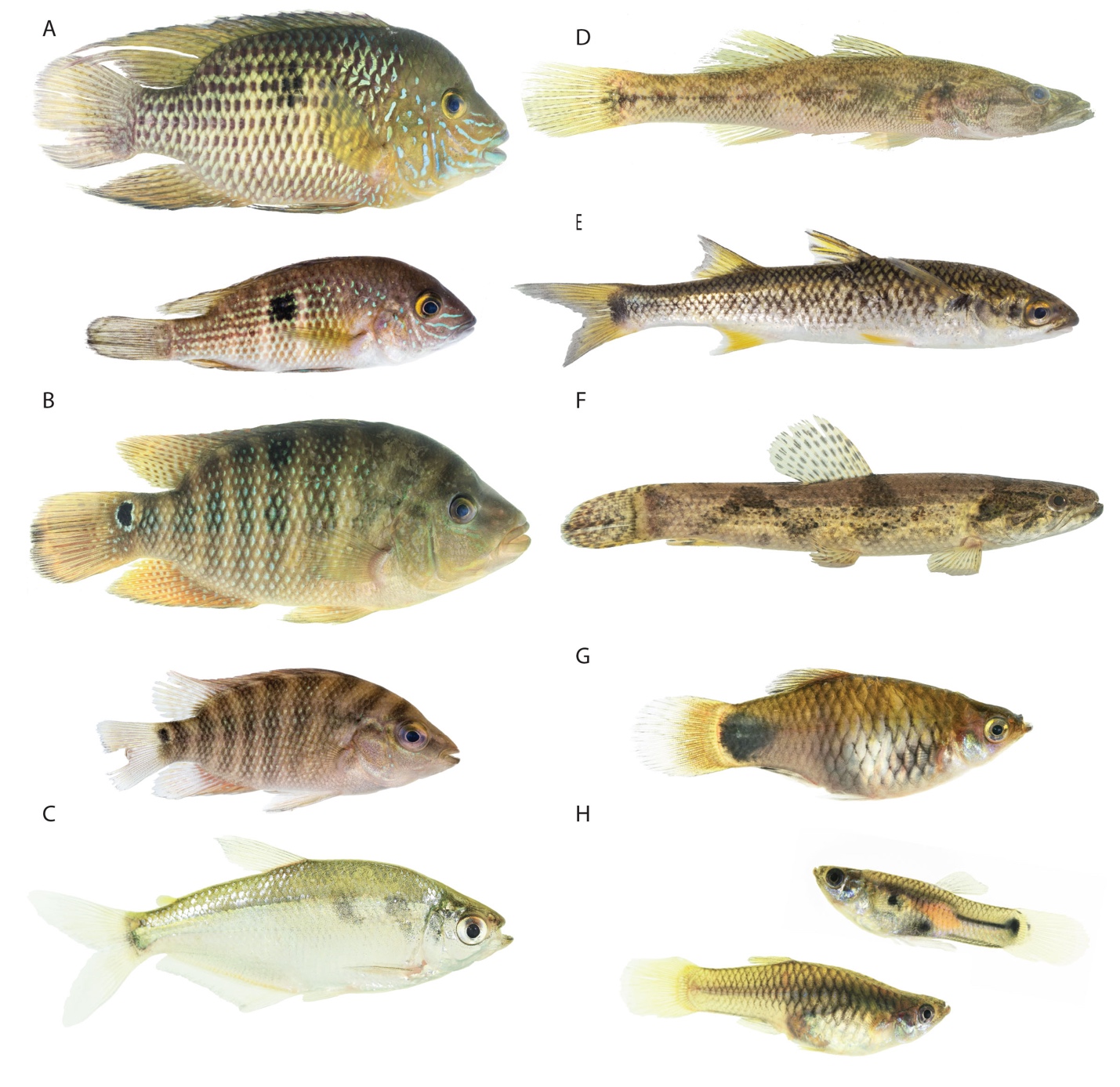


**Figure S2.** Freshwater fish of the Cube River basin. A) *Andinoacara rivulatus* (adult and juvenile) B *Mesoheros festae* (adult and juvenile) C) *Eretmobrycon festae*, D) *Gobiomurus maculatus*, E) *Agonostomus monticola*, F) *Hoplias microlepis*, G) *Xiphophoruss maculatus*, H) *Poecilia reticulata* (male top, female below)*.* Photos: courtesy of Karla Barragán


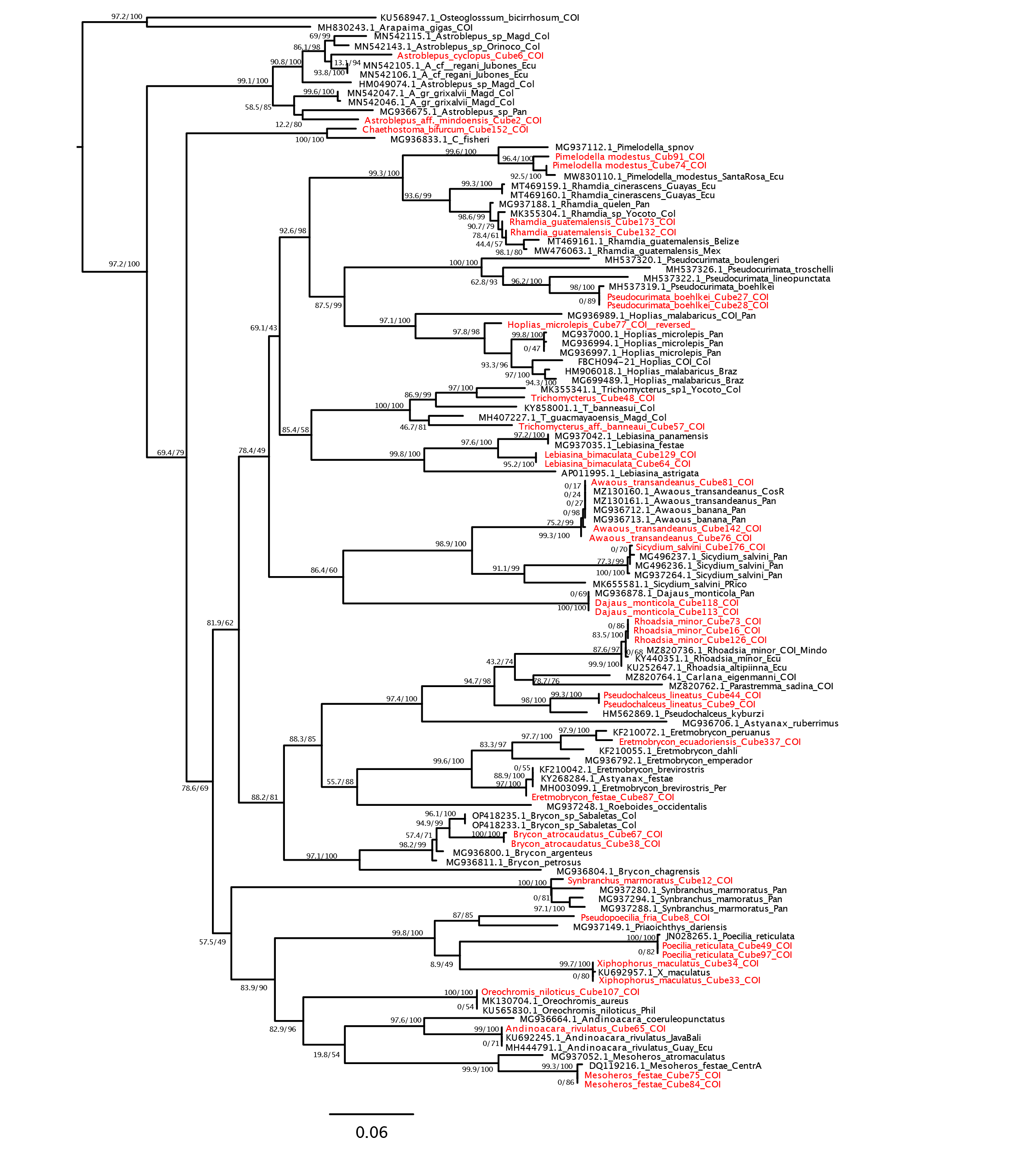


**Figure S3:** Maximum likelihood phylogenetic tree based on partial c oxidase subunit I (COI) sequences (675 bp), including fish species from the Cube River basin and closely related species from South America. Node statistical support is shown as: SH-aLRT support (%) / ultrafast bootstrap support (%).

**Figure S4**: Maximum likelihood phylogenetic tree based on partial 16S rRNA (16S) sequences (663 bp) including fish species from the Cube River basin and closely related species from South America. Node statistical support is shown as: SH-aLRT support (%) / ultrafast bootstrap support (%).


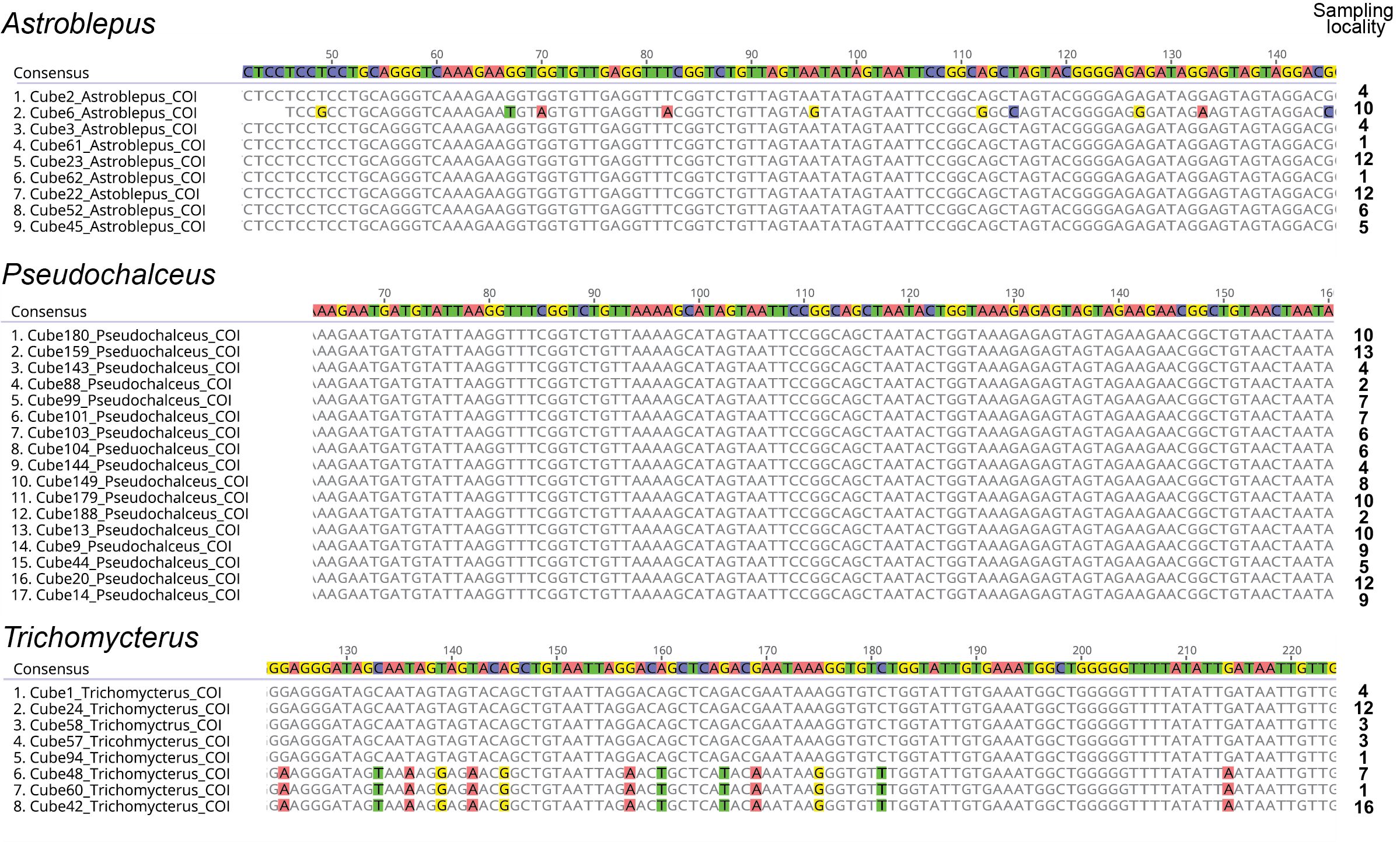


**Figure S5:** Image of sequence-alignments of three fish genera from the Cube River Basin.

**References:**

Albornoz-Garzón, J.G., Villa-Navarro, F.A., 2017. Range extension of the invasive fish Xiphophorus maculatus (Günther, 1866) (Cyprinodontiformes: Poeciliidae) in the upper Magdalena river basin, Colombia. Check List 13, 1–7.

Arguello, P., Jimenez-Prado, P., Sanchez-Duarte, P.&, 2016. Astroblepus cyclopus. The IUCN Red List of Threatened Species. https://doi.org/10.2305/IUCN.UK.2016-1.RLTS.T49830313A66185862.en

Bussing, W.A., 1996. Sicydium adelum, a new species of gobiid fish (Pisces: Gobiidae) from Atlantic slope streams of Costa Rica. Rev. Biol. Trop 44, 819–825.

Chabarria, R.E., Pezold, F., 2013. Phylogeography and historical demography of Sicydium salvini in the eastern Pacific. Ichthyol Res 60, 353–362. https://doi.org/10.1007/s10228-013-0363-x

Cucalón, R. V., Valdiviezo-Rivera, J., Jiménez-Prado, P., Navarrete-Amaya, R., Shervette, V.R., Torres-Noboa, A., Wierzal, N., Karpan, K.C., Borders, T., Calle, P., Lujan, N.K., Aguirre, W.E., 2022. Phylogeography of the Chocó Endemic Rainbow Characin (Teleostei: Rhoadsia). Ichthyology & Herpetology 110, 138–155. https://doi.org/10.1643/i2020092

Hernández, C.L., Ortega-Lara, A., Sánchez-Garcés, G.C., Alford, M.H., 2015. Genetic and Morphometric Evidence for the Recognition of Several Recently Synonymized Species of Trans-Andean Rhamdia (Pisces: Siluriformes: Heptapteridae). Copeia 103, 563–579. https://doi.org/10.1643/CI-14-145

Jiménez-Prado, P. Peces en ríos bajos del noroccidente ecuatoriano, un ejemplo de cauces con la presencia de especies invasoras, 2021. 1st ed. In: Jiménez-Prado P, Valdiviezo-Rivera. J, editors. Diversidad de peces en Ecuador Ictiología ecuatoriana Serie especial I. 1st ed. Red Ecuatoriana de ictiología, Pontificia Universidad Católica del Ecuador Sede Esmeraldas, Universidad Tecnológica Indoamérica, Instituto Nacional de Biodiversidad.. p. 168.

Jiménez-Prado, P., Aguirre, W., Laaz Moncayo, E., Navarrete Amaya, R., Nugra Salazar, F., Rebolledo Monsalve, E., Zárate Hugo, E., Torres Noboa, A., Valdiviezo Rivera, J., 2015. Guía de Peces para Aguas Continentales en la Vertiente Occidental del Ecuador. Pontificia Universidad Católica del Ecuador Sede Esmeraldas (PUCESE); Universidad del Azuay (UDA) y Museo Ecuatoriano de Ciencias Naturales (MECN) del Instituto Nacional de Biodiversidad, Esmeraldas, Ecuador, p. 415.

Lujan, N.K., Meza-Vargas, V., Astudillo-Clavijo, V., Barriga-Salazar, \ Ramiro, López-Fernández, H., 2015. A Multilocus Molecular Phylogeny for Chaetostoma Clade Genera and Species with a Review of Chaetostoma (Siluriformes: Loricariidae) from the Central Andes. Copeia 103, 663–701.

Lyons, T., 2020. Hoplias microlepis, Dientón.

Malato, G., Shervette, V.R., Amaya, R.N., Rivera, J.V., Salazar, F.N., Delgado, P.C., Karpan, K.C., Aguirre, W.E., 2017. Parallel body shape divergence in the Neotropical fish genus Rhoadsia (Teleostei: Characidae) along elevational gradients of the western slopes of the Ecuadorian Andes. PLoS One 12. https://doi.org/10.1371/journal.pone.0179432

Mattox, G.M.T., Bifi, A.G., Oyakawa, O.T., 2014. Taxonomic study of Hoplias microlepis (Günther, 1864), a trans-Andean species of trahiras (Ostariophysi: Characiformes: Erythrinidae). Neotropical Ichthyology 12, 343–352. https://doi.org/10.1590/1982-0224-20130174

McMahan, C., Elias, D., 2020. Awaous transandeanus, Chupapiedras.

NatureServe, Lyons, T.J., 2019. Dajaus monticola.

Navarrete-Amaya R, Shervette VR, Vélez D, Aguirre WE. Patrones biogeográficos y taxonómicos de los peces de la vertiente occidental del Ecuador, 2021. 1st ed. In: Jiménez-Prado P, Valdiviezo-Rivera. J, editors. Diversidad de peces en Ecuador Ictiología ecuatoriana Serie especial I. 1st ed. Red Ecuatoriana de ictiología, Pontificia Universidad Católica del Ecuador Sede Esmeraldas, Universidad Tecnológica Indoamérica, Instituto Nacional de Biodiversidad. p. 168.

Perdices, A., Bermingham, E., Montilla, A., Doadrio, I., 2002. Evolutionary history of the genus Rhamdia (Teleostei: Pimelodidae) in Central America. Mol Phylogenet Evol 25, 172–189. https://doi.org/10.1016/S1055-7903(02)00224-5

Román–Valencia, C., Ruiz–C, R.I., Taphorn B, D.C., Jiménez–Prado, P., García–Alzate, C.A., 2015. A new species of Bryconamericus (Characiformes, Stevardiinae, Characidae) from the Pacific coast of northwestern Ecuador, South America. Anim Biodivers Conserv 38, 241–252. https://doi.org/10.32800/abc.2015.38.0241

Terán, G.E., Benitez, M.F., Mirande, J.M., 2020. Opening the Trojan horse: phylogeny of Astyanax, two new genera and resurrection of Psalidodon (Teleostei: Characidae), Zoological Journal of the Linnean Society.

Wijkmark, N., Kullander, S.O., Barriga Salazar, R.E., 2012. Andinoacara blombergi, a new species from the río Esmeraldas basin in Ecuador and a review of A. rivulatus (Teleostei: Cichlidae), Ichthyol. Explor. Freshwaters.
